# Y-shaped LVIS Stent-assisted Coiling of Anterior Communicating Artery Aneurysms: A Case Series

**DOI:** 10.7759/cureus.4271

**Published:** 2019-03-19

**Authors:** Samer Zammar, Georgios A Maragkos, Scott Simon

**Affiliations:** 1 Neurosurgery, Penn State Health Milton S. Hershey Medical Center, Hershey, USA; 2 Neurosurgery, Beth Israel Deaconess Medical Center, Boston, USA

**Keywords:** aneurysm, stent, lvis

## Abstract

Intracranial neurovascular stents are primarily used in stent-assisted coil embolization in the treatment of intracranial aneurysms. Recent studies suggest that a variety of these stents can be used for their flow diverting effects to obliterate intracranial aneurysms. In our series, we present two patients with ruptured intracranial aneurysms who presented with recurrent aneurysm that was successfully treated with double Low Profile Visualized Intraluminal Support Junior (LVIS Jr.) stent placement.

## Introduction

The use of flow diverters has been approved for wide-necked, complex and dissecting aneurysms that involve the parent artery and that could be challenging to treat with stent-assisted coil embolization [1-3]. The Low Profile Visualized Intraluminal Support Junior (LVIS^®^ and LVIS^®^ Jr., Microvention-Terumo, Tustin, CA, USA) are approved by the U.S. Food and Drug Administration (FDA) for the endovascular treatment of unruptured, wide-necked, saccular intracranial aneurysms [4]. While they are approved for stent-assisted coil embolization, their flow diverting properties remain to be validated in the clinical setting. We present two cases wherein Jr. stents were used in a crossing fashion to provide flow diversion for intracranial aneurysms. This may suggest that the LVIS and LVIS Jr. stents could be used for their flow diverting effect, expanding on the indications of the pipeline embolization device (PED) and to treat aneurysms in the posterior circulation.

## Case presentation

Case 1

**Figure 1:**
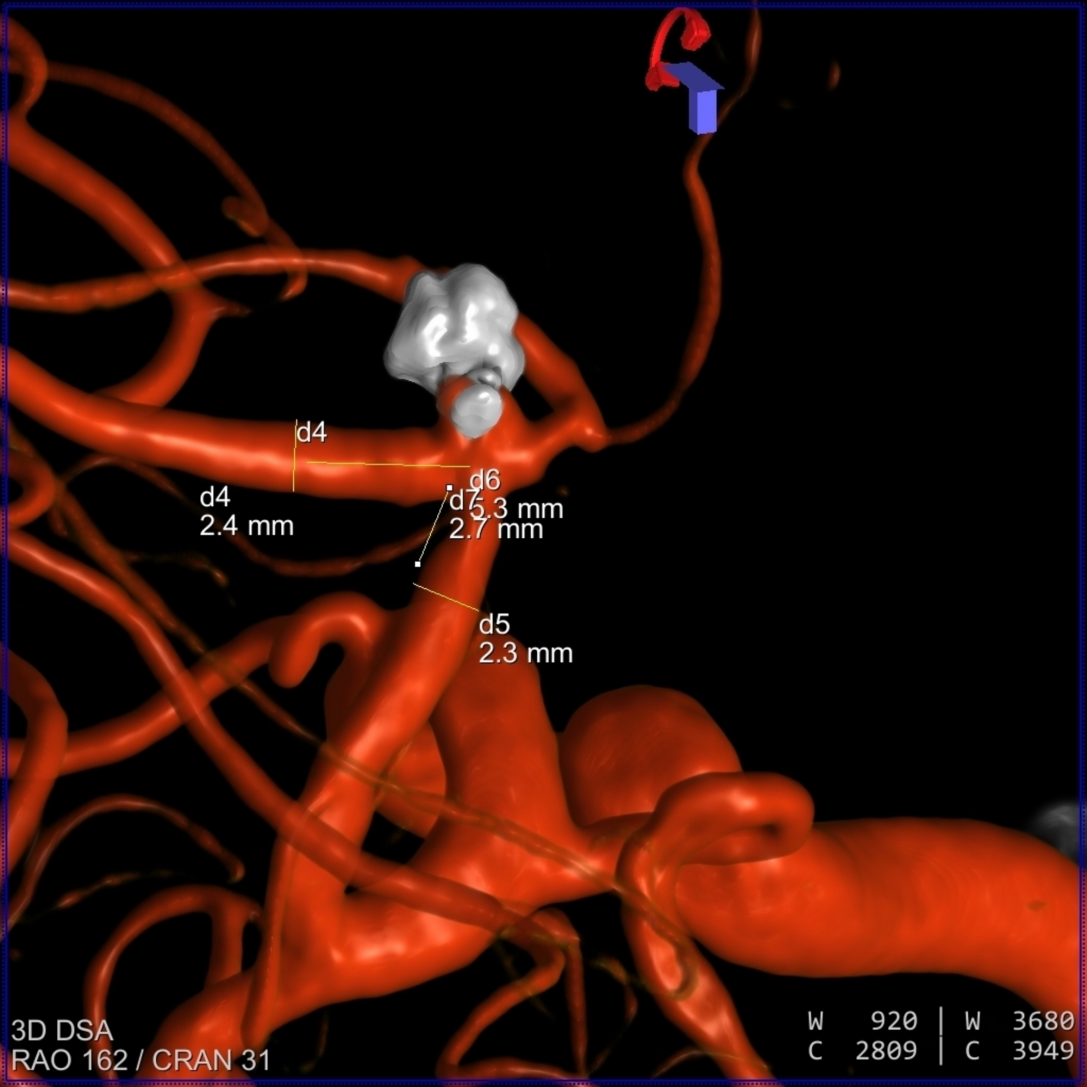
3-D angiogram showing the recanalization of a posterior 2-mm lobe in the anterior communicating artery aneurysm. The figure also shows the length of the corresponding arteries.

A 66-year-old male was previously admitted to our hospital with a subarachnoid hemorrhage (SAH) secondary to a ruptured anterior communicating artery (Acom) aneurysm. He underwent coil embolization of the aneurysm, which initially showed no residual filling; however, subsequent recanalization of a posterior 2-mm lobe was detected on one-year follow-up (Figure 1).

The decision was made to retreat the patient.

**Figure 2:**
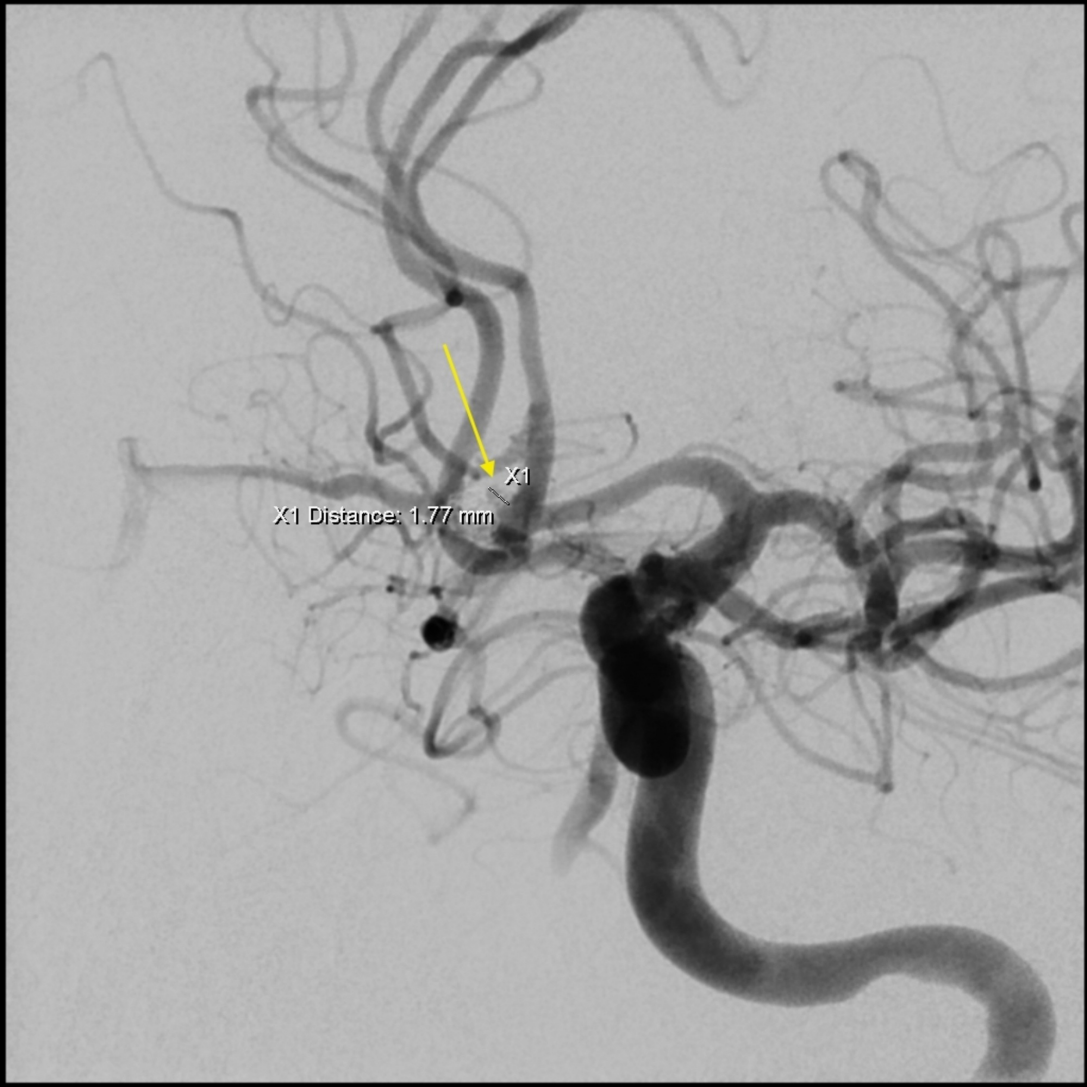
Angiogram of left internal carotid artery revealing an aneurysm remnant, 2 x 1.9 mm in size with one coil loop.

The right femoral artery was accessed using the standard fashion. Next, with the catheter in the left internal carotid artery (ICA), a rotational angiogram was performed, revealing an aneurysm remnant, 2 x 1.9 mm in size, with one coil loop (Figure 2).

A "Y" stent-assisted coil embolization was utilized for treatment. In the first patient, the operator deemed that there is no safe corridor or technique to place the coils in the recurrent aneurysm. In the second case, the initial case was initially thought that it would be stent assisted coil embolization. The coil was attempted to be placed; however, it kept prolapsing so the decision was to leave the stents.

**Figure 3:**
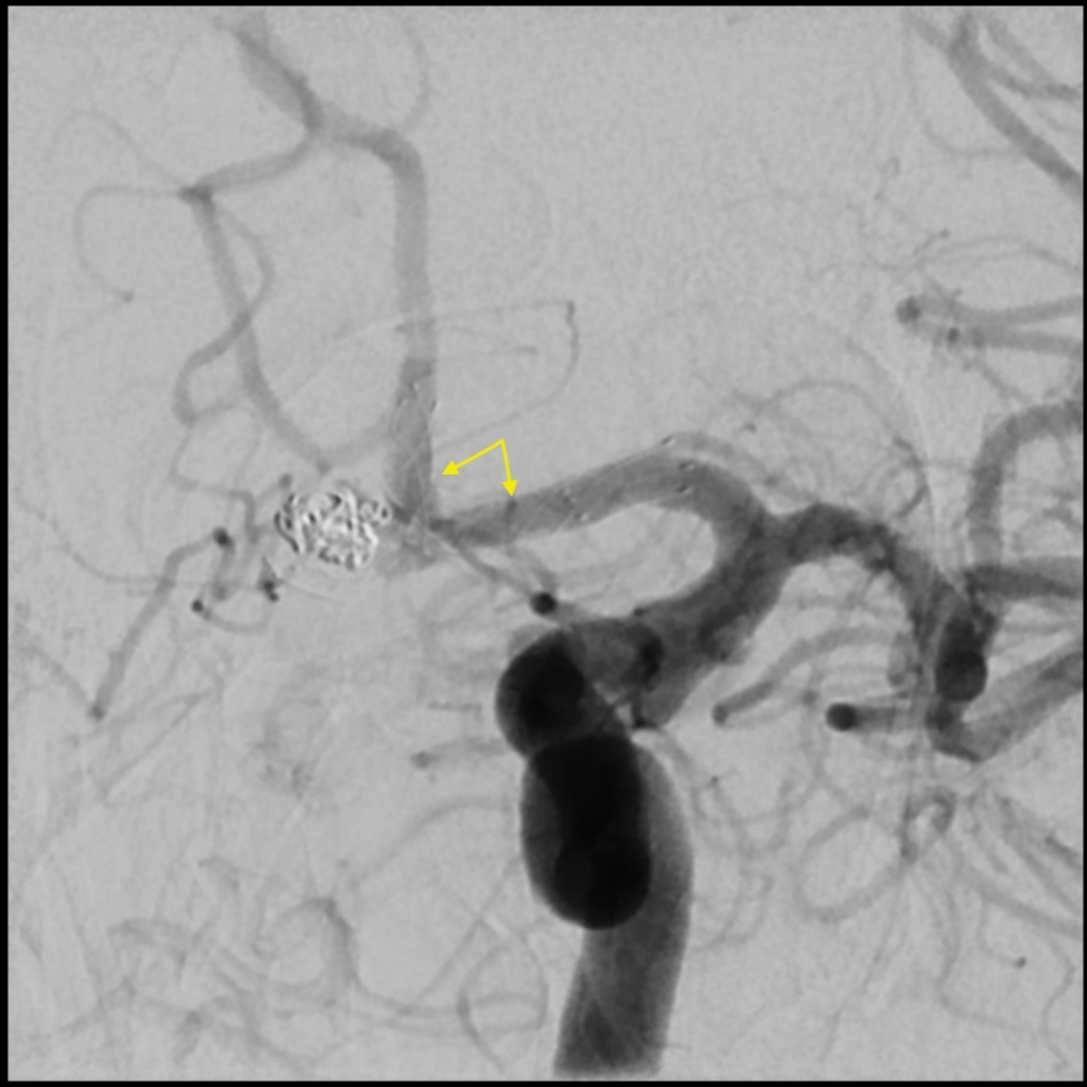
Follow-up left ICA angiogram after deploying the LVIS Jr. stent revealing no filling of the aneurysm at this point. ICA: Internal Carotid Artery; LVIS Jr.: Low-profile Visualized Intraluminal Support Junior.

The diagnostic catheter was replaced with a guiding catheter. A microcatheter was advanced over a microwire into the intracranial ICA, then the left A1 segment and finally across the Acom to the right A2. Then, the wire was removed, and a 2.5 x 23-mm LVIS® Jr. braided coil-assist stent was placed from the right A2 to the left A1. Subsequently, the stent pusher wire was removed and the microwire was replaced and advanced to the ipsilateral A2 segment. With the catheter in this position, the wire was removed, and an LVIS® Jr. 2.5 x 17-mm stent was placed. Follow-up angiographic runs revealed no filling of the aneurysm at this point (Figure 3).

A femoral artery closure device was deployed at the end of the case.

**Figure 4:**
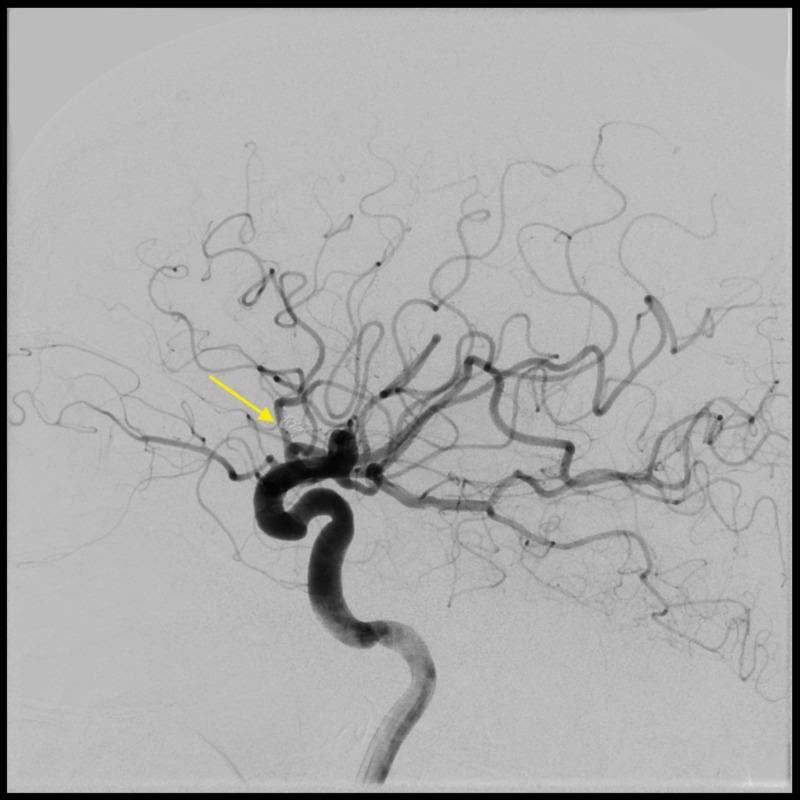
Lateral carotid angiogram showing no aneurysm filling on six-month follow-up.

Postoperatively, he was transferred to the neurological intensive care unit (ICU) and was continued on aspirin and clopidogrel. He was neurologically intact, and no aneurysm filling was noted on six-month follow-up (Figure 4).

Case 2

A 41-year-old female with a history of a ruptured Acom aneurysm treated with balloon-assisted coiling, presented with worsening headaches and a new Acom aneurysm was found in her three-year follow-up. She was noted to have intermittent residual right-sided weakness after her initial subarachnoid hemorrhage. Her home medications were aspirin and clopidogrel. Surgical and non-surgical options were discussed with the patient, including observation, and she opted for endovascular treatment of her new aneurysm.

**Figure 5:**
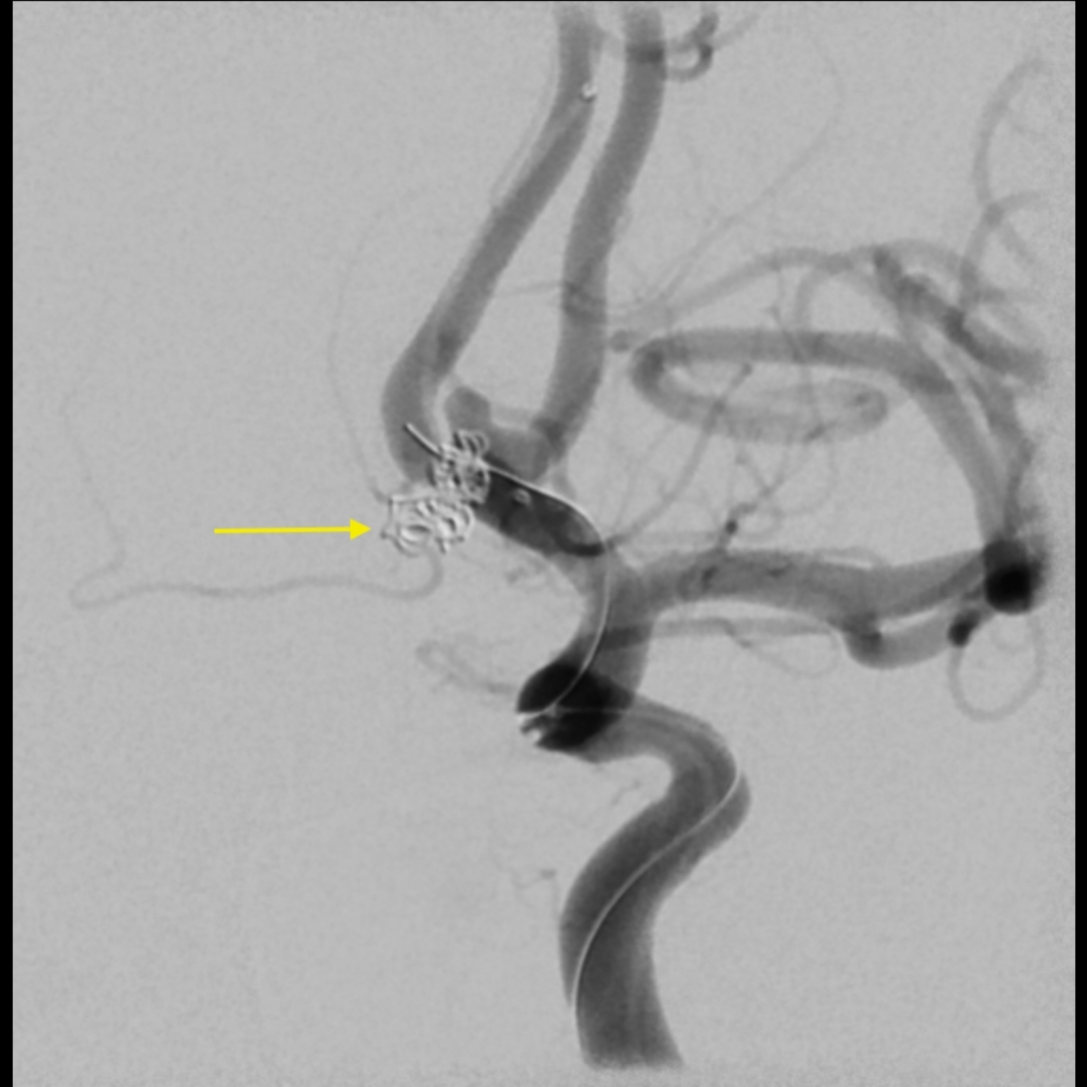
Left ICA angiogram revealing a wide-necked Acom aneurysm. ICA: Internal carotid artery; Acom: Anterior communicating artery.

The patient was brought to the neurosurgical operative suite. She was placed in the supine position on the operating table; both legs were prepped and draped in sterile fashion. The left common carotid and left ICA were sequentially catheterized, contrast injected, and a rotational digitally subtracted angiogram (DSA) performed, which revealed a wide-necked Acom aneurysm (Figure 5), a suitable candidate for stent-assisted coil embolization.

At this point, the diagnostic catheter was swapped out for a guiding catheter and a Headway® microcatheter, (MicroVention Inc., Aliso Viejo, CA, USA) over a microwire, was advanced from the left A1 segment into the right A2. Next, a microcatheter was advanced over a microwire into the aneurysm. An attempt was made to place a 2 mm x 3 mm coil, but it kept prolapsing into the parent artery; therefore, a 2.5 mm x 23 mm LVIS® Jr. stent was placed into the right anterior cerebral artery, extending into the left A1. Again, coil placement was attempted, but it kept herniating this time into the left A2. Therefore, the Headway® microcatheter was reinserted into the guiding catheter and advanced into the A1 on the left and then the A2 on the left. A second 2.5 mm x 23 mm LVIS® Jr. stent was placed.

At this point, an attempt was made to place a 2 mm x 3 mm coil, but it kept prolapsing and the location of these prolapsed loops was unclear, therefore the coil was withdrawn. Next, we tried a 1.5 mm x 3 mm coil and had the same problem. Finally, we tried a 1.5 mm x 2 mm and encountered the same issue. While attempting to adjust this coil, the microcatheter came out of the aneurysm. We were unable to cross the two stents to return to the aneurysm.

A final angiographic run revealed slow filling in bilateral anterior cerebral arteries. Therefore, an intra-arterial dose of abciximab was given, and the patient was started on IV abciximab drip. Follow-up angiogram showed significant improvement, although not back to baseline, and the procedure was terminated.

**Figure 6:**
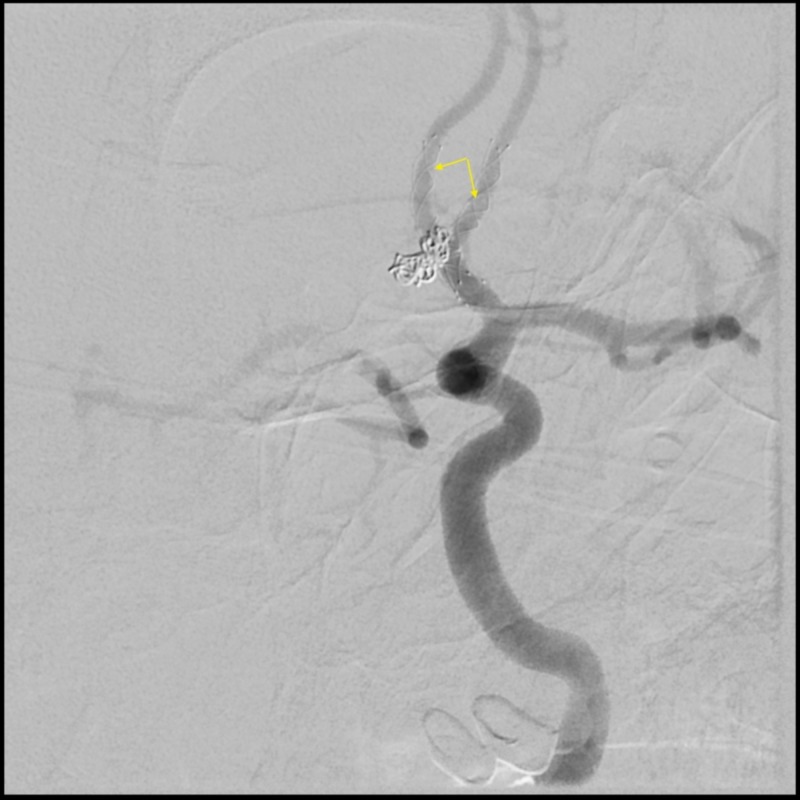
Six-month post-operative angiogram showing complete obliteration of the aneurysm and the double LVIS Jr. stents deployed in a Y fashion. LVIS Jr.: Low-profile Visualized Intraluminal Support Junior

Postoperatively, the patient was maintained on aspirin and ticagrelor. On postoperative day 2, she complained of worsening unrelenting headache, which she rated as 8/10 in intensity. Computed tomography (CT) scan revealed a 4.0 x 3.5 cm left frontal lobe acute intraparenchymal hemorrhage. She was managed conservatively in the neurologic ICU and made a significant recovery. She was discharged home on postoperative day 7, neurologically intact. A six-month post-operative angiogram showed complete obliteration of the aneurysm (Figure 6).

## Discussion

Flow diversion, an alteration of the blood flow within the aneurysm, via a stent subsequently leads to thrombosis and obliteration of the aneurysm [5]. Eventually, neointimal proliferation occurs across the neck of the aneurysms to contribute to the final obliteration. An alternative option to create a flow diversion at the neck of the aneurysm is to use bridging stents at the neck of the aneurysm. The LVIS stent has a metal density higher than the conventional Enterprise (Cordis Neurovascular, Miami, FL, USA) [6]. Compared to PED, the LVIS and LVIS Jr. have 23% and 18% degree of metal coverage as opposed to 30-35% [6-8]. The LVIS and LVIS Jr. stents have been approved for use with embolic coils to treat wide neck (neck ≥ 4 mm, dome-to-neck ratio < 2:1) aneurysms, arising from parent vessels, with diameters ranging from 2 to 4.5 mm. Typically, the LVIS stent is used in vessel diameters of 3-4.5 mm and the LVIS Jr. is used for vessel diameters of 2.5-3 mm [9]. The latter has a low profile nature and can be deployed through a 0.017-inch inner diameter microcatheter, which simplifies accessing small vessels [10].

The FDA has approved the use of the PED to treat large or giant wide-necked intracranial aneurysms in the internal carotid artery from the petrous to the superior hypophyseal segments [11]. Wang et al. were able to show that a single LVIS stent can provide more wall shear stress reduction than a set of two Enterprise stents. Furthermore, significant reduction in wall shear stress and velocity was observed with double LVIS stents, superior to a single pipeline device [6]. Thus, a double LVIS stent resulted in better flow diverting effect than a PED. While the flow diverting effect has been reported for LVIS stent, no current report exists discussing flow diversion with the LVIS Jr. stent. Our case series demonstrates successful obliteration of recurrent aneurysms with double LVIS Jr. stents. Importantly, we demonstrate that crossing, not just overlapping LVIS Jr. stents, may display flow-diverting effects; and, although this is well short of proof, clinicians may consider this for the treatment of bifurcation or posterior circulation aneurysms that are difficult to coil.

## Conclusions

In this case series we demonstrate successful obliteration of recurrent aneurysms using a set of two LVIS Jr. stents. We agree that the anti-platelets might have contributed to the hemorrhage. We thought treating the aneurysm has an acceptable risk to benefit ratio compared to not treating. Of course we attempted coiling first and we could not get the coil to stay in the aneurysm. It kept prolapsing. Our study showcases the flow-diverting properties of this construct. The LVIS Jr. stent may be a reasonable flow-diverting stent option for wide-necked recurrent intracranial aneurysms, after coil embolization. This option could be an alternative treatment for recurrent aneurysms, particularly when a pipeline, stent-assisted coiling or open surgical treatment is not feasible. Further clinical validation studies are needed to compare the flow-diverting effects of these stents and the pipeline embolization device.

## References

[1] Geyik S, Yavuz K, Yurttutan N, Saatci I, Cekirge HS (2013). Stent-assisted coiling in endovascular treatment of 500 consecutive cerebral aneurysms with long-term follow-up. AJNR Am J Neuroradiol.

[2] Kühn AL, Hou SY, Perras M, Brooks C, Gounis MJ, Wakhloo AK, Puri AS (2015). Flow diverter stents for unruptured saccular anterior circulation perforating artery aneurysms: safety, efficacy, and short-term follow-up. J Neurointerv Surg.

[3] Becske T, Kallmes DF, Saatci I (2013). Pipeline for uncoilable or failed aneurysms: results from a multicenter clinical trial. Radiology.

[4] Poncyljusz W, Bilinski P, Safranow K (2015). The LVIS/LVIS Jr. stents in the treatment of wide-neck intracranial aneurysms: multicentre registry. J Neurointerv Surg.

[5] Eller JL, Dumont TM, Sorkin GC (2014). The Pipeline embolization device for treatment of intracranial aneurysms. Expert Rev Med Devices.

[6] Wang C, Tian Z, Liu J (2016). Flow diverter effect of LVIS stent on cerebral aneurysm hemodynamics: a comparison with Enterprise stents and the Pipeline device. J Transl Med.

[7] Kojima M, Irie K, Fukuda T, Arai F, Hirose Y, Negoro M (2012). The study of flow diversion effects on aneurysm using multiple enterprise stents and two flow diverters. Asian J Neurosurg.

[8] Roszelle BN, Gonzalez LF, Babiker MH, Ryan J, Albuquerque FC, Frakes DH (2013). Flow diverter effect on cerebral aneurysm hemodynamics: an in vitro comparison of telescoping stents and the Pipeline. Neuroradiology.

[9] Cho YD, Sohn CH, Kang HS (2014). Coil embolization of intracranial saccular aneurysms using the Low-profile Visualized Intraluminal Support (LVIS) device. Neuroradiology.

[10] Gupta M, Cheung VJ, Abraham P (2019). Low-profile Visualized Intraluminal Support Junior device for the treatment of intracranial aneurysms. Cureus.

[11] Zammar SG, Buell TJ, Chen CJ (2018). Outcomes after off-label use of the pipeline embolization device for intracranial aneurysms: a multicenter cohort study. World Neurosurg.

